# The Way to a Human’s Brain Goes Through Their Stomach: Dietary Factors in Major Depressive Disorder

**DOI:** 10.3389/fnins.2020.582853

**Published:** 2020-12-07

**Authors:** Janine Aly, Olivia Engmann

**Affiliations:** ^1^Faculty of Medicine, Friedrich Schiller Universität, Jena, Germany; ^2^Institute for Human Genetics, Jena University Hospital, Jena, Germany

**Keywords:** major depressive disorder, dietary factors, antidepressants, vitamins, fatty acids, caffeine, minerals

## Abstract

Globally, more than 250 million people are affected by depression (major depressive disorder; MDD), a serious and debilitating mental disorder. Currently available treatment options can have substantial side effects and take weeks to be fully effective. Therefore, it is important to find safe alternatives, which act more rapidly and in a larger number of patients. While much research on MDD focuses on chronic stress as a main risk factor, we here make a point of exploring dietary factors as a somewhat overlooked, yet highly promising approach towards novel antidepressant pathways. Deficiencies in various groups of nutrients often occur in patients with mental disorders. These include vitamins, especially members of the B-complex (B6, B9, B12). Moreover, an imbalance of fatty acids, such as omega-3 and omega-6, or an insufficient supply with minerals, including magnesium and zinc, are related to MDD. While some of them are relevant for the synthesis of monoamines, others play a crucial role in inflammation, neuroprotection and the synthesis of growth factors. Evidence suggests that when deficiencies return to normal, changes in mood and behavior can be, at least in some cases, achieved. Furthermore, supplementation with dietary factors (so called “nutraceuticals”) may improve MDD symptoms even in the absence of a deficiency. Non-vital dietary factors may affect MDD symptoms as well. For instance, the most commonly consumed psychostimulant caffeine may improve behavioral and molecular markers of MDD. The molecular structure of most dietary factors is well known. Hence, dietary factors may provide important molecular tools to study and potentially help treat MDD symptoms. Within this review, we will discuss the role of dietary factors in MDD risk and symptomology, and critically discuss how they might serve as auxiliary treatments or preventative options for MDD.

## MDD – a Major Health Challenge in Modern Societies

Anhedonia, feelings of guilt and worthlessness, circadian alterations and a lack of motivation are only some symptoms of major depressive disorder (MDD; Krishnan and Nestler, 2010; Edgar and Mcclung, 2013). In 2016, the global prevalence of MDD amounted to 268.2 million people worldwide and accounted for 44.2 million life-years lost to disability (Vos et al., 2017). Major depressive disorder is the main cause for suicide and therefore potentially lethal (Bebbington, 2001). The MDD-related loss in productivity at work costs governments annually billions of dollars [e.g., Switzerland: 8.2 billion Euros (2008) (Tomonaga et al., 2013); United States: 210.5 billion dollars (2010) (Greenberg et al., 2015)]. Major depressive disorder is now one of the largest categories of health care expenditure, in part due to patients, which are resistant to currently available treatments (Mrazek et al., 2014). In contrast to many formerly life-threatening diseases, which have been successfully controlled due to hygiene, vaccination and antibiotics, the risk for MDD is on the rise in many modern societies (Hidaka, 2012). Hence, the research field of MDD is becoming increasingly recognized.

Throughout the years, various risk factors for MDD were described. Besides a genetic predisposition, multiple environmental risk factors have been determined. These include traumatic events in early or adult life and chronic stress (Mann and Currier, 2006). Hence, a complex interplay between social, psychological and genetic factors seems to be responsible for MDD.

Perhaps due to the multifactorial nature of this illness, a large population of MDD patients does not fully respond to current pharmacological antidepressant treatments (Krystal et al., 2019), which moreover take weeks to reach their full efficacy (Harmer et al., 2017). Nevertheless, the choice in treatment options has been greatly expanded during the last decades. Despite a good amount of serendipity in antidepressant research, over the last years, various treatments have been developed based on functional hypotheses of MDD: Medications that increase the amount of monoamines, suppress an overactive hypothalamic-pituitary-adrenal (HPA) axis or anti-inflammatory drugs are used to counteract MDD (Ferrari and Villa, 2017), albeit more unconventional paths such cholinergic and opioid signaling are under investigation as well (Papakostas and Ionescu, 2015). Although those antidepressants evoke a response at the molecular level within hours, it often takes weeks before a therapeutic effect takes place. A possible alternative, which is mainly used in therapy-resistant cases, lies in the fast-acting compound ketamine (Krystal et al., 2019). This drug, originally used as a sedative and abused as a psychoactive substance, has an anti-depressant effect that occurs within hours and lasts for several days. Consequently, it is a fast-acting and highly effective drug against MDD. However, due to its abuse potential, the use of ketamine is limited and not practicable as a long-term treatment (Andrade, 2017).

Alternatively, there are non-pharmaceutical interventions available. Light therapy, sleep restriction, as well as psycho-therapeutic interventions such as interpersonal therapy and cognitive behavioral therapy can cause some improvements in MDD (Markowitz and Weissman, 2004; Golden et al., 2005; Anthes, 2014; Murphy and Peterson, 2015). Indeed, cognitive behavioral therapy has been shown to be as effective as pharmacological interventions (Weitz et al., 2015). Other non-pharmacological interventions in use for MDD are electroconvulsive therapy, trancranical magnetic stimulation and vagus nerve stimulation, all of which can be effective in refractory cases (O’Reardon et al., 2006). However, some of these treatments come at a considerably larger cost to healthcare providers, require invasive protocols or remain stigmatized (O’Reardon et al., 2006; Ross et al., 2019). In consequence, despite their health-benefits, non-pharmacological interventions are often not sufficiently available.

Interestingly, despite accumulating evidence for a link between MDD and nutrition, dietary factors as potential pharmacological tools for MDD treatment have been largely overlooked. Hence, in this review we will make a point, that more effort should be invested in this direction: Deficiencies in numerous nutrients, including vitamins, minerals or fatty acids, are more commonly observed in patients with mental disorders. By correcting dietary deficiencies, both behavioral changes and an improvement in mood may be achieved (Rao et al., 2008). Furthermore, preliminary evidence suggests that supplementation with certain dietary factors might improve MDD-symptoms even in non-deficient populations. Additionally, non-vital nutritional factors such as caffeine may positively affect MDD-symptomology (Lucas, 2011). Supplementation with dietary factors may be a safe, cost-effective and easily implementable therapeutic approach. Moreover, dietary factors provide well-characterized single-molecule tools to unravel previously unknown molecular pathways underlying MDD in animal models. In this review, we will discuss the role of selected dietary factors in MDD and we will critically examine how they might serve as an adjunctive treatment or preventative option for MDD.

## Depression – an Illness With Many Causes

The brain areas affected in MDD are largely known. These comprise regions involved in cognitive and emotional processing, including the prefrontal cortex, hippocampus, hypothalamus and amygdala as well as the brain reward system (nucleus accumbens, ventral tegmental area, habenula). Nevertheless, the exact pathophysiological origin of the disease remains unclear. Up to now, various hypotheses about the molecular cause of MDD have been established:

1.) The *monoamine hypothesis* is one of the best-known theories on the molecular origins of MDD: The underlying assumption is that a depletion of monoamines is responsible for MDD-symptoms. Monoamines such as norepinephrine (NE), serotonin (5-HT), and dopamine all play a crucial role in the coordination of mood, motivation, and circadian rhythms – processes, which are often affected during MDD (Hamon and Blier, 2013). Several studies have provided causal evidence that an insufficient supply or an increased degradation of monoamines, and dysregulation of subsequent signal transduction pathways, cause MDD-like symptoms (Meyer et al., 2006; Prins et al., 2011; Ferrari and Villa, 2017).
2.) G*rowth factors* are associated with MDD as well. In particular, the brain-derived neurotrophic factor (BDNF) is strongly correlated with antidepressant action (Bjorkholm and Monteggia, 2016). Brain-derived neurotrophic factor is widely expressed in the central nervous system and is important for neuronal maturation, but also for synapse formation and synaptic plasticity. The activity of BDNF is regulated by the cyclic AMP response element binding protein (CREB), which binds to its promoter region. Brain-derived neurotrophic factor modulates tropomyosin receptor kinase B receptors (TRKB), thereby influencing neurotransmission and synaptic plasticity. A transcriptional dysregulation of BDNF and TRKB has been commonly observed in MDD (Dwivedi et al., 2003; Qi et al., 2015; Bjorkholm and Monteggia, 2016) and in suicide completers (Maussion et al., 2014). This suggests that the TRKB-pathway may be an interesting target for possible treatment approaches. Lesser known growth factors implicated in MDD are fibroblast growth factor, vascular endothelial growth factor, insulin-like growth factor and glial cell-line derived neurotrophic factor (Levy et al., 2018).
3.) Another hypothesis implicates the *hypothalamus-pituitary-adrenal (HPA) axis* and specifically addresses the link between chronic stress and MDD-risk (Pariante and Lightman, 2008). Through a hormonal cascade, stress increases the secretion of adrenal glucocorticoids. Glucocorticoids are involved in the control of neuronal survival, neurogenesis, synaptic plasticity and hippocampal size, linking brain function and stress (McEwen, 2017). Intriguingly, glucocorticoids can inhibit their own release via a negative feedback loop, which is thought to be impaired in MDD (Pariante and Lightman, 2008). Chronic stress can lead to a disruption of this negative feedback loop. The result is a frequently observed overactivation of the HPA axis in MDD-patients (Bhagwagar et al., 2003; Bao and Swaab, 2010).
4.) Furthermore, there is evidence for a link between *inflammation* and MDD (Miller et al., 2009). Analysis of peripheral blood from MDD-patients revealed elevated levels of inflammatory biomarkers, including cytokines, chemokines, and adhesion molecules (Howren et al., 2009). These molecules can cross the blood–brain barrier, where they interact with different brain regions involved in monoamine synthesis, neuroendocrine regulation, and neuronal plasticity, which are heavily implicated in MDD (Ferrari and Villa, 2017). Since inflammation affects monoamines and the HPA axis, an interaction between many different factors is likely to contribute to MDD.
5.) Cellular damage due to excitotoxicity, changed gene expression, chronic stress (e.g., due to nitric oxide) or elevated inflammation in the brain can also be observed during *aging*. Symptoms that are associated with MDD, including cognitive decline, fatigue, or sleep disturbances, often occur in aged populations, especially in the context of neurodegenerative diseases. Furthermore, MDD patients have a shorter average life expectancy. This connection led to the hypothesis that MDD is a condition of accelerated brain aging (Heuser, 2002; Wolkowitz et al., 2010; Kinser and Lyon, 2013). Postmortem examination of the brains of MDD patients revealed anatomical changes consistent with this idea, including a reduction in gray matter volume (Grieve et al., 2013; Zhao et al., 2014), neuronal loss (Banasr et al., 2011), and impaired cerebral blood flow (Burrage et al., 2018). On a molecular level, MDD patients tend to show a reduced telomere length (Monroy-Jaramillo et al., 2018), altered DNA methylation (Peña and Nestler, 2018) and accumulated damage in mtDNA (Kasahara and Kato, 2018), which all have been implicated in aging (Booth and Brunet, 2016). However, it is currently unclear, whether these alterations contribute to MDD or are the result of it.
6.) MDD can be, at least in part, understood as a metabolic disorder. There is a bidirectional comorbidity between MDD and obesity (Milaneschi et al., 2019). Above-mentioned alterations in the HPA-axis and inflammation as well as genetics may be shared risk factors for both, MDD and metabolic changes. However, more direct metabolic regulators may affect MDD as well. For instance, the feeding hormone leptin has antidepressant-like effects in mouse models (Yamada-Goto et al., 2011; Garza et al., 2012), while deletion of the leptin receptor in certain brain areas can induce resistance to common antidepressants (Guo and Lu, 2014). There may also be an association between insulin resistance and MDD in patients (Kan et al., 2013). The microbiome, which is critical in both, regulating nutrient uptake and fighting inflammation, appears to be affected in MDD patients as well (Jiang et al., 2015; Zhernakova et al., 2016; Lin et al., 2017). However, secondary effects due to antidepressant medications need to be ruled out when interpreting those studies. Given those contributing factors, it is intriguing to speculate that MDD may be a manifestation of a metabolic syndrome. Interestingly exercise, a main regulator of metabolism, has been repeatedly shown to ameliorate MDD symptoms (Carek et al., 2011).
7.) Twin studies suggest that MDD is only 37% heritable (Kendler, 2001). Hence, there is a strong environmental component to this disorder. Epigenetic mechanisms can modify chromatin without affecting the DNA-sequence and are known to integrate environmental risk factors and genetic propensity to ultimately affect gene transcription. There is accumulating evidence that a variety of chromatin modifications is altered both, in peripheral tissue (typically blood), and postmortem in the brains of MDD patients (Penner-Goeke and Binder, 2019).

DNA methylation is thought to be the most stable and long-lasting chromatin modification. Hence, MDD risk factors such as early life trauma may alter DNA methylation patterns to shape disease risk later in life (Matosin et al., 2017). In monozygotic twin studies, DNA methylation patterns were associated with MDD risk later in life (Palma-Gudiel et al., 2020). Of the many DNA methylation changes described in MDD, changes in the BDNF gene have been most often replicated (Chen et al., 2017; Penner-Goeke and Binder, 2019).

Not only DNA, but histones, too, can be methylated – albeit by a different enzymatic machinery. Of the plethora of histone residues, changes in the repressive marks H3K4me3 and H3K27me3 have been described in MDD patients and suicide victims (Ernst et al., 2009; Chen et al., 2011; Fiori et al., 2012). Moreover, histone acetylation changes have been observed postmortem at the enhancer mark H3K14ac (Covington et al., 2009). Accordingly, changes in histone deacetylase enzymes HDAC2 and SIRT1 have been detected in postmortem brain tissues of MDD patients (Covington et al., 2009; Kishi et al., 2010; Ledford, 2015). Rodent models of MDD have shown an even larger number of changed histone marks, suggesting that there may be a specific histone foot print induced by chronic stress (Nestler et al., 2015). Besides “classical” epigenetic modifications on histones and DNA, a large number of micro-RNA changes in peripheral tissue has been implicated in MDD. Of these, miR-132 has most often been replicated (Yuan et al., 2018). Furthermore, a reduced telomere length has been observed in patients with MDD (Henje Blom et al., 2015; Monroy-Jaramillo et al., 2018) and bipolar depression (BD; Squassina et al., 2017; Powell et al., 2018). Telomeres are relevant to maintain genome stability and their shortening is typically associated with aging in mitotic cells. Furthermore, mutations in mitochondrial DNA have been detected in patients suffering from MDD and BD (Kato, 2017; Czarny et al., 2018).

The epigenetics field is highly dynamic. New histone modifications such as dopaminylation and homocysteinylation are still being discovered (Zhang et al., 2018; Lepack et al., 2020). It is very likely that additional chromatin marks that are just being described or are currently still unknown, play a role in MDD as well.

All of the MDD hypotheses mentioned above are interconnected. For instance, changed DNAme in the BDNF pathway connects epigenetic and growth factor aspects (Bakusic et al., 2017), while increased inflammation and oxidative stress can lead to epigenetic changes such as reduced telomere length and mtDNA mutations (Czarny et al., 2018).

## Vitamins and Their Role in Depression

### Vitamin B6 Is an Essential Puzzle Piece for Neurotransmitter Production

Pyridoxine, pyridoxal, and pyridoxamine are three related, naturally occurring isoforms that are grouped together under the name of vitamin B6. They can be obtained from a variety of food, including meat, dairy products, grains, nuts, vegetables, and certain fruits. Once absorbed in the small intestine, all isoforms can be converted into pyridoxal 5′-phosphate (PLP), the active metabolite of B6, which modulates more than 150 enzymatic reactions within the body. In consequence it is involved in several processes related to mental function and mood as well (Hvas et al., 2004; Mikkelsen et al., 2016).

**FIGURE 1:**
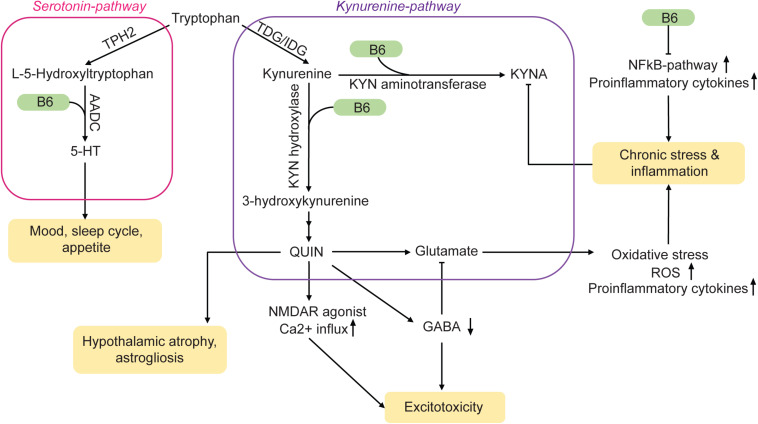
Vitamin B6 acts on MDD-related pathways via a variety of mechanisms. TDG/IDG, tryptophan dioxygenase or indoleamine dioxygenase; KYN, kynurenine; QUIN, quinolonic acid; ROS, reactive oxygen species; TPH2, tryptophan hydrolase 2; AADC, amino acid decarboxylase; KYNA, kynurenic acid. Arrows: permissive effects, cross bars: inhibitory effects.

The production of serotonin (5-hydroxytryptamine, 5-HT), a neurotransmitter that is depleted during MDD, takes place primarily in the raphe nuclei of the brain. Although it is possible to ingest 5-HT with the diet, the molecule itself cannot cross the blood-brain barrier. However, tryptophan (TRP), the precursor of 5-HT, can enter the brain. Two enzymes within serotonergic neurons are then responsible for the conversion of TRP into 5-HT: Hydroxylation of TRP is induced by tryptophan hydroxylase 2 (TPH2), followed by decarboxylation through aromatic L-amino acid decarboxylase (AADC) (Figure 1). Pyridoxal 5′-phosphate acts as a cofactor for AADC and is hence crucial for the synthesis of 5-HT in the brain. Therefore, a vitamin B6 deficiency could lead to reduced 5-HT levels and may thus be a potential risk factor for MDD (Höglund et al., 2019; Mikkelsen et al., 2016). Furthermore, low levels of PLP may be associated with stress (McCarty, 2000). Accordingly, supplementation with both, vitamin B6 and TRP, increases 5-HT neurotransmission (Shabbir et al., 2013).

Vitamin B6 supplementation improved MDD-symptoms in an aged cohort and vitamin B6 levels generally correlated with MDD severity (Moore et al., 2019). Furthermore, lower vitamin B6 intake correlated with disease severity in female MDD-patients (Kafeshani et al., 2019). The diagnosis of vitamin B6 deficiency in MDD patients and the subsequent change in diet could therefore serve as a treatment complementary to drug therapy.

Vitamin B6 is not only part of the 5-HT synthesis pathway, it also plays a role in the kynurenine pathway (Figure 1). Pyridoxal 5′-phosphate serves as a co-factor for both, kynurenine aminotransferase and kynurenine hydroxylase. The products of this pathway are the neuroprotective metabolite kynurenic acid (KYNA) and the neurotoxic substance quinolinic acid (QUIN). Normally, the effects of KYNA and QUIN are well balanced. However, during chronic stress or inflammation, the equilibrium can be shifted towards QUIN. Being an agonist of *N*-methyl-D-aspartate (NMDA) receptors, high concentrations of QUIN can induce excitotoxicity (Guillemin, 2012) and may contribute to the neural damage often observed in MDD-brains (Miura et al., 2008; Meier et al., 2016). Elevated glutamate concentrations can also lead to oxidative stress and mitochondrial damage via an increase in nitric oxide synthases (nNOS, iNOS) (Dawson et al., 1991), which can contribute to inflammatory processes. An upregulation of inflammatory mediators is not only induced by QUIN but also occurs in the absence of vitamin B6 (Ueland et al., 2017).

Pyridoxal 5′-phosphate is a co-factor for a variety of enzymes that affect the homocysteine pathway (see below) (Wu and Lu, 2012). Vitamin B6 may therefore indirectly alter chromatin marks such as DNA- and histone methylation as well as chromatin structure.

Furthermore, vitamin B6 is an important metabolite in the production of gamma amino butyric acid (GABA) out of its precursor glutamate (McCarty, 2000; Jung et al., 2019). A decreased turnover of GABA due to vitamin B6 deficiency could therefore result in a lack of inhibitory feedback in the brain. This mechanism could further amplify the damage caused by increased glutamate levels.

NFκB, a transcription factor involved in the production of cytokines and cell survival, is influenced by vitamin B6 as well. It modulates the immune response of macrophages and is suppressed in the presence of PLP (Yanaka et al., 2005). The resulting inflammatory response activates the kyurenine pathway further (Ueland et al., 2017). Therefore, vitamin B6 has key functions in monoamine synthesis and is involved in the regulation of the immune response to various stressors. A misregulation of these processes due to insufficient supply can lead to 5-HT depletion and increased inflammation, which links vitamin B6 deficiency to a variety of risk factors for MDD.

Prophylactic intake of vitamin B6 did not reduce the risk of MDD among older women in a 7-year longitudinal study (Okereke et al., 2015). However, in this study, potential prior deficiencies have not been investigated, which impedes the interpretation of the data. In contrast, vitamin B6 appeared to rapidly ameliorate symptoms such as low mood in young MDD-patients when the disease was already present, even though in this study, deficiencies have not been assessed either (Tsujita et al., 2019). Furthermore, the beneficial properties of vitamin B6 have been demonstrated in a variety of non-deficient rodent studies. For instance, vitamin B6 improved behavioral measures in forced swim and sucrose preference tests in the dexamethasone mouse model of depression (Mesripour et al., 2019). Additionally, when co-administered, vitamin B6 enhanced the effect of antidepressants clomipramine and venlafaxine but not fluoxetine in the forced swim test (Mesripour et al., 2017). However, further research is required to make more precise predictions on the efficacy of vitamin B6 supplements in MDD. For instance, in human cohorts, an undiagnosed vitamin B6 deficiency or an interaction with other risk factors such as stress and comorbid diseases should be taken into consideration when designing future studies. In mouse models, the effect of vitamin B6 on the susceptibility to chronic stress should be investigated, paying special attention to chronic versus fast-acting effects of vitamin B6. Furthermore, the impact of vitamin B6 on manic states in BD is insufficiently investigated. For instance, a case report suggests that vitamin B6, at least in combination with other substances, may induce secondary mania (Chaturvedi and Upadhyaya, 1988). However, it appears that vitamin B6 may have a positive effect on lithium-induced tremor in patients (Dias Alves et al., 2017).

### Two Main Players Within the One Carbon Metabolism – Vitamin B9 and B12

**FIGURE 2:**
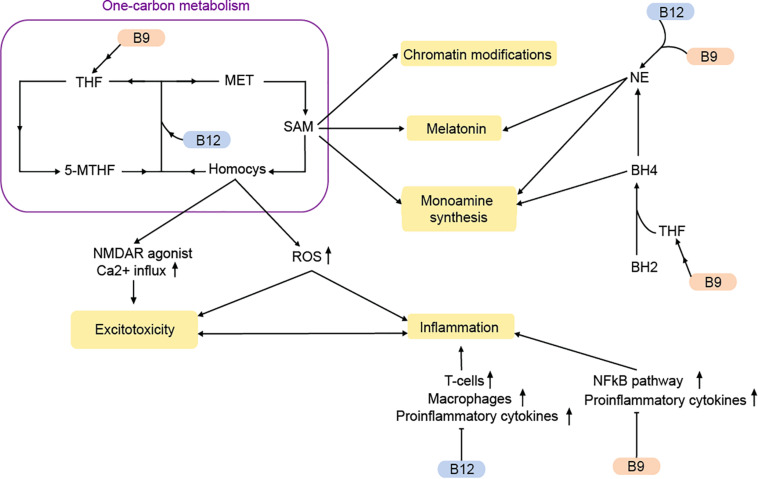
Vitamins B9 and B12 regulate MDD-pathways via the one-carbon metabolism and beyond. MET, methionine; Homocys, homocysteine; NE, norepinephrine; THF, tetrahydrofolate; 5-MTHF, 5-methyltetrahydrofolate; ROS, reactive oxygen species; SAM, S-adenosylmethionine; BH2, dihydrobiopterin; BH : tetrahydrobiopterin. Arrows: permissive effects, cross bars: inhibitory effects.

Two other members of the vitamin B-complex are often associated with MDD. Vitamin B9 (folate) is primarily obtained from plant-based nutrients, while vitamin B12 (cobalamin) is only synthesized by bacteria and enriched across the food chain in animal products and certain algae or fungi. Together they are two interdependent nutrients, which are involved in the regulation of various essential processes, including the methionine cycle, the one-carbon metabolism and monoamine synthesis (Figure 2).

The methionine cycle, which is responsible for the synthesis of methionine and the universal methyl donor S-adenosylmethionine (SAM), heavily depends on vitamin B9 and B12. Methionine is produced through the methylation of homocysteine (Froese et al., 2019). This reaction is catalyzed by the methionine synthase, which uses vitamin B12 as a co-factor and 5-methyltetrahydrofolate (5-MTHF), a primary biological active form of vitamin B9 as methyl-group donor. During vitamin B9/12 deficiencies, 5-MTHF conversion to tetrahydrofolate (THF) is decreased. At the same time there is an accumulation of the metabolite homocysteine (Mikkelsen et al., 2016). Although homocysteine is an important precursor for the production of methionine, SAM, and THF, elevated concentrations of homocysteine cause cerebral damage and a decline in cognitive abilities and memory (Obeid and Herrmann, 2006; Moore et al., 2018). Being an agonist of the NMDA receptor, homocysteine can increase postsynaptic Ca^2+^ levels and thereby contribute to excitotoxicity (Lipton et al., 1997).

Elevated homocysteine levels can also promote oxidative stress via the production of reactive oxygen species (ROS; Djuric et al., 2018). The result is a disruption in energy supply, which can lead to neuronal damage and an increased inflammatory response (Obeid and Herrmann, 2006). Correspondingly, a vitamin B9 deficiency causes an increased expression of pro-inflammatory cytokines via homocysteine (Mikkelsen et al., 2017). Inflammation is affected by vitamin B9/12 via other pathways as well. The NFκB pathway is suppressed by vitamin B9 (Au-Yeung et al., 2006). Moreover, weak evidence suggests that vitamin B12 stimulates the proinflammatory interleukin-6 production in rats (Scalabrino et al., 2002).

A deficiency in vitamin B9 and B12 not only causes elevated homocysteine levels and increased inflammation in the brain, but also reduces the amount of available SAM (as measured in a reduced SAM/SAH ratio). The methyl donor SAM is required for the methylation of DNA, proteins and neurotransmitters, thereby controlling their transcription, structure and activity. Hence, abnormal SAM-levels can affect transcription, chromatin structure and DNA repair (De Berardis et al., 2016). Accordingly, SAH is inhibitor of methyltransferases (Berger and Sassone-Corsi, 2016). Notably, there is accumulating evidence that SAM may have antidepressant effects (De Berardis et al., 2016; Karas Kuželièki, 2016). Rats fed with a diet rich in methyl-donors show improved depressive-like behaviors (McCoy et al., 2016)

Folate deficiency is associated with increased levels of DNA methyltransferases and histone methyltransferases (Ghoshal et al., 2006). Furthermore, supplementation with a variety of nutraceuticals including folate, cobalamin, choline, and L-methionine, inhibited HDAC1 in rodents (Cho et al., 2014). Unfortunately, few studies have investigated the impact of single vitamins on chromatin marks, especially not in human peripheral tissue. An intriguing new link between the one-carbon metabolism and chromatin may be the posttranslational modification homocysteinylation, which has been detected in neurons on histone 3 lysine 79 (Zhang et al., 2018). It’s relevance to mental disorders is currently not known.

Besides the one-carbon metabolism, vitamin B9 and B12 can affect monoamine synthesis. Tetrahydrobiopterin (BH4) is a major cofactor in the conversion of various amino acids to 5-HT, dopamine, and NE. Tetrahydrobiopterin is the limiting agent in the synthesis, while being also extremely susceptible to oxidation. The oxidized form of BH4, known as dihydrobiopterin (BH2) is reconverted into BH4 with the help of dihydrofolate reductase, an enzyme requiring THF as a cofactor for its reaction. During vitamin B9 deficiency, there is lower BH2 recycling and monoamine production (Miller, 2008). This could result in reduced levels of 5-HT and dopamine that are often observed in MDD (Miller, 2008; Ramaekers et al., 2016).

With the help of SAM, NE is converted into melatonin, a main hormonal regulator of circadian rhythms, which is often disrupted in MDD (Mendoza, 2019). Dysregulation of the noradrenergic system due to vitamin B9/12 deficiency may therefore contribute to MDD-symptoms through a variety of mechanisms.

In summary, vitamin B9 and vitamin B12 are involved in many mechanisms that are impaired in MDD. The synthesis of monoamines, the regulation of the immune response, chromatin modifications as well as the removal of metabolic by-products with neurotoxic effects are disrupted during vitamin B9/12 deficiencies. Both interact and influence one another. This, however, also results in studies that are often insufficiently distinguishing between the effects of each vitamin on its’ own.

Human studies on vitamin B9 generally support its’ role in MDD. For instance, a large meta-analysis shows that vitamin B9 deficiency is associated with higher MDD risk, even after controlling for confounding factors (Gilbody et al., 2007). Vitamin B9 deficiency also occurs more often in treatment-resistant patients and correcting this deficiency can aid in MDD-treatment (Coppen and Bolander-Gouaille, 2005; Fava and Mischoulon, 2009; Mikkelsen et al., 2016). The link between vitamin B12 and Major depressive disorder is even more evident. Vitamin B12 deficiency leads to symptoms of MDD, and can be diagnosed in up to one third of MDD patients (Mikkelsen et al., 2016). Accordingly, chronic supplementation with injected vitamin B12 improved MDD-symptoms in two large cohorts (Walker et al., 2012; Syed et al., 2013). Notably, chronic supplementation with either, vitamin B9 or vitamin B12, may improve MDD-symptoms particularly in men (Murakami et al., 2008; Gougeon, 2014).

In rodents, vitamin B12 deficiency induces MDD-like symptoms (Ghosh et al., 2018). Notably, chronic supplementation with methyl donors including vitamin B9 and vitamin B12 improved depression-like behaviors in a non-deficient rodent model of early life stress (Paternain et al., 2016). Chronic vitamin B9 supplementation on its’ own prevented stress-induced depressive-like effects in mice (Rosa et al., 2014). Interestingly, an acute dose of vitamin B9 was sufficient to prevent the detrimental effects of acute stress on behavior and hippocampal biomarkers of MDD in mice as well (Budni et al., 2013). Furthermore, a single acute dose of vitamin B12 in non-deficient mice ameliorated molecular and behavioral effects reminiscent of depression induced by a combination of chronic and acute stress (Trautmann et al., 2020a). This suggests, that vitamin B9 and B12 may potentially have fast-acting antidepressant effects, at least in rodents. Hence, the underlying pathways should be explored further to reveal potentially undescribed fast-acting antidepressant pathways.

Serum folate levels are reduced in patients with BD as well (Hsieh et al., 2019). Furthermore, BD patients may have elevated homocysteine levels during the manic episode (Permoda-Osip et al., 2013). Accordingly, it has been suggested that folate as an adjunct therapy with regular medication may be beneficial to treat manic phases in BD patients (Behzadi et al., 2009; Nierenberg et al., 2017). In rats, too, a combination of chronic folate and lithium chloride improved behavioral measures in the metamphine model of mania (Menegas et al., 2020). However, in a prospective study with people at familial risk for mood disorders, no effect of folate on disease was observed but folate supplementation caused a slight delay in disease onset (Sharpley et al., 2014). Unfortunately, despite being otherwise rigorously designed, this study, too, did not assess any initial deficiencies. To our knowledge, no hypomania was induced by folate or vitamin B12 supplementation.

## Trace Minerals and MDD-Risk

### Magnesium May Improve Mood Through the Gut

The bivalent cation magnesium (Mg^2+^) is an important modulator of various processes in the human body. It serves as a cofactor in over 300 different reactions, including DNA replication, transcription and translation (Schwalfenberg and Genuis, 2017). Obtained from foods such as nuts, seeds, grains, and green leafy vegetables, Mg^2+^ is essential for healthy brain function. Accordingly, Mg^2+^ is implicated in a variety of illnesses (Volpe, 2013).

**FIGURE 3:**
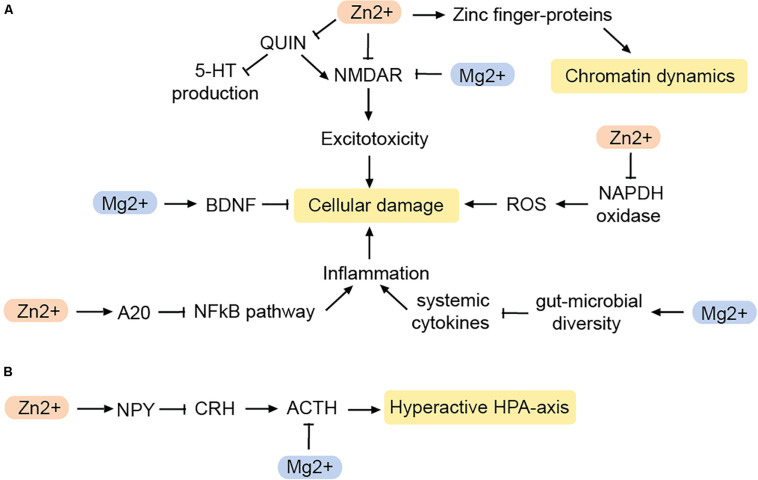
Impact of zinc and magnesium on MDD-related pathways. **(A)** Pathways reducing cellular damage. **(B)** Pathways that inhibit a hyperactive HPA-axis. BDNF, brain-derived neurotrophic factor; NPY, neuropeptide Y; CRH, corticotropin-releasing hormone; ACTH, adenocorticotropic hormone; QUIN, quinolonic acid; ROS, reactive oxygen species. Arrows: permissive effects, cross bars: inhibitory effects.

Mg^2+^ blocks the NMDAR in a voltage-dependent manner. Hence, in the brain, Mg^2+^ deficiency can lead to excitotoxicity and ROS production (Murck, 2013; Figure 3A). Indeed, the action of Mg^2+^ on the glutamatergic system has been likened to that of the glutamatergic regulator ketamine, which has fast-acting antidepressant effects (Górska et al., 2019). Accordingly, both ketamine and Mg^2+^ evoke similar downstream changes such as increased expression of eukaryotic elongation factor 2 and BDNF (Slutsky et al., 2010; Pochwat et al., 2015), and both have similar effects on slow wave sleep in humans (Murck, 2013).

Mg^2+^ deficiency also acts on the brain indirectly via the gut-brain-axis. The gut microbiome produces hormones and neuro-active molecules and has a major impact on the immune system. Notably, there is an interplay between microbiome composition and MDD (Bastiaanssen et al., 2020). Mg^2+^ deficiency disturbs the composition of the gut microbiome in mice (Pyndt Jørgensen et al., 2015), while Mg^2+^ supplementation increases the microbial diversity in the gut (Crowley et al., 2018). Mg^2+^ induced changes in the microbiome can also affect the levels of circulating cytokines, and therefore increase the risk for systemic inflammation during Mg^2+^ deficiency (Wang et al., 2018). An increase in the inflammatory markers C-reactive protein (CRP), IL-6, and TNFα can contribute to neuronal damage and defective myelination, and may contribute to the cognitive symptoms in some depressed patients (Cubała and Landowski, 2014). Since glutamate-induced ROS can trigger an immune response as well, Mg^2+^ deficiency can alter the immune response by two different mechanisms.

However, not only the immune response is modified by Mg^2+^, but also the stress response (Figure 3B). Mg^2+^ controls the HPA axis by reducing the release of adrenocorticotropic hormone (ACTH) as well as modulating the sensitivity to it (Wang et al., 2018). As a result, Mg^2+^ may prevent the hyperactivation of the HPA axis often seen in MDD-patients.

Mg^2+^ plays a role in enzymatic reactions that mediate the maintenance of DNA structure. In particular, the role in maintaining telomere integrity is well described. Here, Mg^2+^ appears to be particularly necessary to maintain Lamin B interaction with the chromatin (Maguire et al., 2018). Furthermore, Mg^2+^ may indirectly increase the risk of mtDNA damage by being required for a proper energy metabolism in mitochondria (Zheltova et al., 2016; Maguire et al., 2018).

In rodent studies, Mg^2+^ deficiency induces depressive-like behaviors (Singewald et al., 2004; Spasov et al., 2008), while co-administration of Mg^2+^ potentiates the beneficial effects of common antidepressants (Poleszak et al., 2007). In human studies, there is some evidence for Mg^2+^ deficiency occurring more commonly in MDD patients. However, the effects are heterogeneous (Serefko et al., 2013; Yary et al., 2016; Wang et al., 2018; Górska et al., 2019; Sun et al., 2019; Tarleton et al., 2019). On the other hand, chronic Mg^2+^ supplementation appears to improve MDD symptoms even without a prior diagnosis of Mg^2+^ deficiency (Tarleton et al., 2017). Intriguingly, improvements may occur much more rapidly than with traditional antidepressant treatment (Eby and Eby, 2006).

While Mg^2+^ supplementation appears to have antidepressive effects, at least in rodents and certain human populations, high doses of Mg^2+^ supplements can have a variety of side effects, so careful assessment of an underlying Mg^2+^ deficiency and proper dosing are key (Van Laecke, 2019).

### The Immunomodulating Mineral Zinc

The second most common trace element in the human body after Mg^2+^ is zinc (Zn^2+^). Although Zn^2+^ can be obtained from plant foods including cereals and legumes, the absorption of the ion can be impaired by the phytic acid present in plants. The bioavailability of Zn^2+^ from crops is therefore often limited (Gibson et al., 2010). The main sources of Zn^2+^ are thus red meat, oysters and crabs. Zn^2+^ is involved in countless enzymatic reactions, both systemically and in the brain (Takeda, 2000). These include pathways that regulate biosynthesis, neurogenesis, antioxidant defense and the immune response (Szewczyk et al., 2011; Wang et al., 2018; Prasad and Bao, 2019).

Intriguingly, there is a strong negative association between MDD and Zn^2+^ levels, which has led to the suggestion to use serum Zn^2+^ levels as a biomarker for affective disorders (Maes et al., 1994; Szewczyk et al., 2011; Siwek et al., 2013; Wang et al., 2018). Correspondingly, Zn^2+^ supplementation generally improves MDD symptoms (Wang et al., 2018) and co-treatment with Zn^2+^ improved antidepressant action in two placebo controlled double-bind studies (Nowak et al., 2003a; Siwek et al., 2010). In various rodent models of depression, both chronic and acute Zn^2+^ supplementation improved depression-like behaviors (Kroczka et al., 2000; Nowak et al., 2003b; Cieślik et al., 2007; Sowa-Kuæma et al., 2008; Wang et al., 2018) and promoted the effects of antidepressants in these models (Szewczyk et al., 2011). It is still unclear how exactly Zn^2+^ is involved in the clinical picture of MDD. However, there is a plethora of hypotheses:

Zn^2+^ is enriched in glutamatergic presynaptic vesicles in the central nervous system (Frederickson et al., 2000). Being an antagonist of the NMDA receptor (Peters et al., 1987) Zn^2+^ may help to reduce excitotoxicity (Figure 3). Zn^2+^ also may have antioxidant effects by inhibiting the enzyme NADPH oxidase, which is responsible for the production of ROS, and generates proteins that act as scavengers for free radicals (Doboszewska et al., 2016). Therefore, Zn^2+^ deficiency results in a greater production of ROS, and increased levels of QUIN. As a result, QUIN as NMDA agonist increases excitotoxicity while at the same time, less TRP is available for 5-HT production (Doboszewska et al., 2016). Zn^2+^ can also counteract the neurotoxicity caused by chronic inflammation via the NFκB-pathway.

A20, a zinc-finger containing protein, is an endogenous inhibitor of the NFκB-pathway. Zn^2+^ leads to an increased production of the A20 and thereby suppresses the formation of pro-inflammatory cytokines (Hongxia et al., 2019). This suggests that Zn^2+^ affects inflammation and oxidative stress in various ways and thus serves as an important regulator of pathways that are known to contribute to MDD.

Zn^2+^ is also involved in the regulation of neuropeptide Y (NPY) (Lee et al., 1998; Levenson, 2003). Neuropeptide Y is released from nerve endings in various regions of the brain, including the hypothalamus, cortex, amygdala, and hippocampus, some of which are associated with MDD. Both, Zn^2+^ and NPY regulate daily food intake (Levenson, 2003), may be of interest in the context of anhedonia as a symptom of MDD as well as co-morbid illnesses such as eating disorders (Shay and Mangian, 2000). Furthermore, NPY controls sleep and is particularly involved in promoting and modulating the REM sleep phase (Dyzma et al., 2010). However, probably the most important aspect linking NPY to MDD is the peptide’s involvement in maintaining the homeostasis of the HPA axis. In the hypothalamus, NPY antagonizes corticotropin-releasing hormone, which normally coordinates the release of ACTH from the pituitary gland (Thorsell, 2010). Therefore, NPY can indirectly inhibit ACTH. Adrenocorticotropic hormone release follows a circadian rhythm and helps prevent the hyperactivation of the HPA axis (Heilig, 2004; Adam and Epel, 2007; Morales-Medina et al., 2010).

Altered levels of NPY and its receptors have been reported in the context of MDD and stress (Morales-Medina et al., 2010). In rodent models, Zn^2+^ deficiency prevents the release of NPY and thus may cause a dysregulation of various physiological functions, including feeding behaviors and the stress response (Levenson, 2003; Reichmann and Holzer, 2016).

Zn^2+^ may also indirectly act as a neuromodulator, although its function in this respect is still poorly understood. Zn^2+^ can be stored in synaptic vesicles and may help facilitate GABA-ergic neurotransmission (Minami et al., 2002). Zn^2+^ can also interact with ZnR/GPR39, a G-protein coupled receptor (Hershfinkel, 2018). Interestingly, ZnR/GPR39 is downregulated in MDD and upregulated by antidepressant treatment (Młyniec et al., 2015). Furthermore, in mice the zinc receptor agonist TC-G 1008 reduces the immobility time in the forced swim test (Starowicz et al., 2019). Activation of ZnR/GPR39 triggers a variety of biochemical pathways associated with cell proliferation, anti-apoptotic properties and neuroplasticity (Hershfinkel, 2018). These include the ERK/MAPK signaling pathway (Holst et al., 2007), the AKT/PI3K signaling pathway (Dong et al., 2016) and the CREB/BDNF pathway (Mlyniec et al., 2015). Hence, ZnR/GPR39 is involved in various mechanisms that are dysregulated in MDD, making it an interesting potential therapeutic target for MDD.

Zn^2+^ is a cofactor for histone deacetylases, the enzymes which remove acetyl groups from histones (Seto and Yoshida, 2014). Some of these, including HDAC2 and SIRT1, are implicated in MDD (Lu et al., 2018; Penner-Goeke and Binder, 2019). Additionally, protein binding to the DNA is faciliated by a so called zinc finger-domain. Accordingly, zinc finger proteins regulate chromatin on a variety of levels (Klug, 2010). They include transcription factors as well as the structural factor CTCF (Phillips and Corces, 2009). Hence, a Zn^2+^ deficiency is likely to fundamentally disturb chromatin dynamics and in consequence transcription.

Taken together, Zn^2+^ may impact on MDD via direct and indirect mechanisms. Correcting Zn^2+^ deficiencies may improve MDD-symptoms and amplify the effects of antidepressant treatment. Studies on the molecular basis of Zn^2+^ action have revealed a bouquet of previously unknown neural pathways and mechanisms. However, Zn^2+^ should only be taken in physiological quantities as overdosing can induce a variety of side effects including neurotoxicity (Salzman et al., 2002; Yang et al., 2013).

## Neuroprotection via Fatty Acids – Omega-3 and Omega-6

The organ with the highest proportion of fatty acids, besides adipose tissue itself, is the brain (Sastry, 1985). Fatty acids are essential for the development and maintenance of the central nervous system, are involved in various biological processes within the brain and play an important role in the stability and structure of membranes (Rao et al., 2008). Of all lipids found in the brain, poly-unsaturated fatty acids (PUFAs) constitute the largest fraction (Sastry, 1985). In this context, the so-called Omega-3 (ω3) and Omega-6 (ω6) PUFAs are of great importance. Members of these two groups cannot be synthesized by the body and must therefore be provided by the diet (Simopoulos, 1991; Gibson and Makrides, 2001). The short-chain precursors of ω3 (α-linoleic acid, ALA) and ω6 PUFAs (linoleic acid, LA) can be obtained from fish, cereals, vegetables or cereal oil. ALA and LA are further converted into docosahexaenoic acid (DHA), eicosapentaenoic acid (EPA), and arachidonic acid (AA), generating the three main long chain PUFAs present in the brain (Thomas Larrieu and Layé, 2018). Here, they serve as signal molecules and are involved in the regulation of membrane fluidity and function, inflammation, the HPA axis and neurogenesis. As soon as the long-chain ω3 and ω6 PUFAs are transported into the brain, the fatty acids are esterified at and attached to neuronal and glial cell membranes (Layé et al., 2018). Through the activation of glutamatergic, serotonergic, cholinergic, or dopaminergic receptors, or through inflammation, PUFAs can be released from the cell membrane (Bazinet and Layé, 2014). In their detached forms, they can act on a variety of receptors, including the G-protein coupled receptor GPR32 and the peroxisome proliferator-activated receptor γ (PPARγ) (Bazinet and Layé, 2014; Marion-Letellier et al., 2016). Peroxisome proliferator-activated receptor agonists have neuroprotective and anti-inflammatory properties (Kapadia et al., 2008) and have been suggested for the treatment of MDD (Colle et al., 2017). Furthermore, ω3 and ω6 PUFAs themselves display anti-oxidative effects and, when metabolized to oxylipins, they are involved in modulating the immune response as well as the activity of the HPA axis (Bazinet and Layé, 2014; Layé et al., 2018).

**FIGURE 4:**
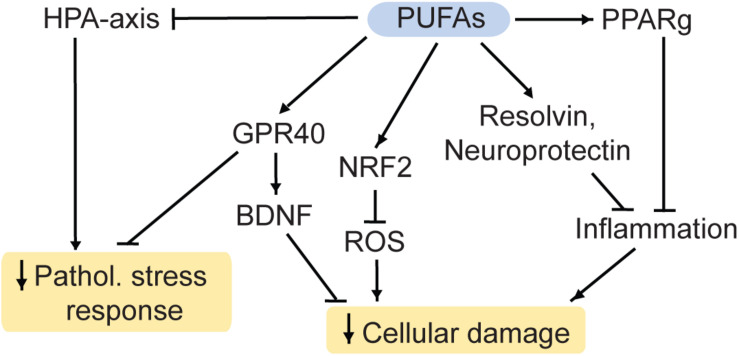
PUFAs reduce cellular damage and the pathological stress response. HPA, hypothalamic-pituitary-adrenal; PUFA, polyunsaturated fatty acid; ROS, reactive oxygen species; Pathol., pathological; PPARg, Proliferator-activated receptor gamma; NRF2, nuclear factor-like 2. Arrows: permissive effects, cross bars: inhibitory effects.

DHA interacts with retinoid X receptors (RXR; Calderon and Kim, 2007) and the beneficial effects of DHA on measures despair are blocked in RXR knockout-mice (Wietrzych-Schindler et al., 2011). Similarly, inhibition or ablation of the DHA receptor GPR40 (Figure 4) is linked to effects of chronic stress and depression-like states in mouse models (Nishinaka et al., 2014; Aizawa et al., 2017, 2018). Additionally, GPR40 regulates BDNF levels in mice (Sona et al., 2018) and primates (Boneva and Yamashima, 2012).

Furthermore, DHA and its metabolite EPA are used for the production of the factors resolvin and neuroprotectin (Bazinet and Layé, 2014). These proteins inhibit the infiltration of immune cells into the brain, the production of inflammatory modulators and they initiate the restoration of damaged neurons (Layé et al., 2018). Therefore, ω3 PUFAs potentially have anti-inflammatory and neuroprotective properties. Furthermore, they have antioxidant properties: ROS can be regulated by EPA and DHA via the nuclear factor-like 2 (NRF2) antioxidant pathway (Bang et al., 2017; Zgórzyńska et al., 2017).

Heavy research has been undertaken to investigate the link between ω3 PUFAs and MDD. There appears to be an inverse relationship between the intake of food rich in ω3 PUFAs and MDD, although the variability between studies warrants more investigation (Grosso et al., 2014; Larrieu and Layé, 2018). For instance, depression rates are generally higher in regions with low fish consumption, a main source of ω3 PUFAs (Grosso et al., 2014). Accordingly, supplementation with ω3 PUFAs may improve MDD-symptoms (Bozzatello et al., 2016; Trebaticka et al., 2020). ω3 PUFAs also appear to improve markers associated with MDD in humans including the HPA axis (Mocking et al., 2013; Thesing et al., 2018) and inflammatory markers (Layé et al., 2018).

In ω3 PUFA deficient rodents, hyperactivity of the HPA axis was observed as well (Morgese et al., 2017; Tang et al., 2018). Conversely, ω3 PUFA supplementation ameliorated behavioral deficits induced by stress in rodent models (Song et al., 2003; Ferraz et al., 2011). In addition, accumulating evidence suggests that in rodents chronic stress-induced depressive-like behaviors and stress-induced molecular alterations in the HPA axis, antioxidant pathways and the gut microbiome may be ameliorated by ω3 supplementation (Larrieu et al., 2014; Pusceddu et al., 2015; Wu et al., 2016; Réus et al., 2018).

Poly-unsaturated fatty acids can regulate chromatin dynamics. For instance, they are ligands to PPARs, which regulate gene expression, chromatin structure, and chromatin modifying enzymes (Yu and Reddy, 2007; Romagnolo et al., 2014). Accordingly, maternal separation and unpredictable maternal stress (MSUS) stimulates ALA/LA and (AA pathways in adult mice (van Steenwyk et al., 2020). The resulting inhibition of PPARs can affect transposable elements and gene expression. These effects may contribute to intergenerational transmission of certain stress-induced disease risks (van Steenwyk et al., 2020). Effects of various PUFA on DNAme have been described as well (Kiec-Wilk et al., 2011; Kulkarni et al., 2011). Various human studies suggest epigenetic effects of PUFAs, too. Mostly these studies supplement with a combination of several PUFAs and are not controlled for confounding factors (González-Becerra et al., 2019).

Interestingly, butyric acid, a short fatty acid produced by the microbiome from dairy products, can inhibit HDACs (Lee et al., 2017; González-Becerra et al., 2019). The derivative sodium butyrate is promoted for treating mood disorders and cognitive deficits (Stilling et al., 2016).

Mixed results were observed in BD patients when dietary PUFA-supplements were administered or in correlative studies with systemically circulating PUFAs (Saunders et al., 2016). Supplementation with omega-3 may improve BD symptoms (Sarris et al., 2012). However, in BD patients, AA metabolism may be increased as shown by postmortem studies (Kim et al., 2011) and by circulating AA plasma levels (Sublette et al., 2007). Furthermore, medication for BD may reduce the AA cascade (Rapoport, 2014). Consistently, an upregulation of the AA cascade, e.g., via supplements, may worsen the illness (Saunders et al., 2016). Hence, the type of PUFA supplement will be crucial for treatment outcome. Accordingly, an imbalance between ω3 and ω6 PUFAs is often associated with depression, perhaps through an increased inflammatory response (Trebaticka et al., 2020).

Taken together, there is increasing consent that ω3 PUFA supplementation promotes positive outcomes for mental health problems including MDD. Combined with other benefits such as a lower risk for cardiovascular disease, high ω3 PUFA content in the diet is likely to generally improve health in human populations. However, supplementation may have to be tailored to the type of depression (MDD/BD) as well as to the antidepressant treatment that is currently being taken by the patient.

## Caffeine’s Underexplored Mood-Elevating Properties

All previously mentioned dietary factors are essential for proper functioning of the human body. Vitamins, minerals and fatty acids are obtained through diet and a deficit contributes to the development of various diseases, including MDD. Treating an underlying deficiency in these factors may help to reduce MDD-symptoms and there is preliminary evidence that some of these dietary factors may have antidepressant properties even in non-deficient populations. However, there are also non-essential dietary factors that can aid in the treatment of MDD. This includes caffeine, the most frequently consumed psychoactive substance in the world (Ferré, 2013).

**FIGURE 5:**
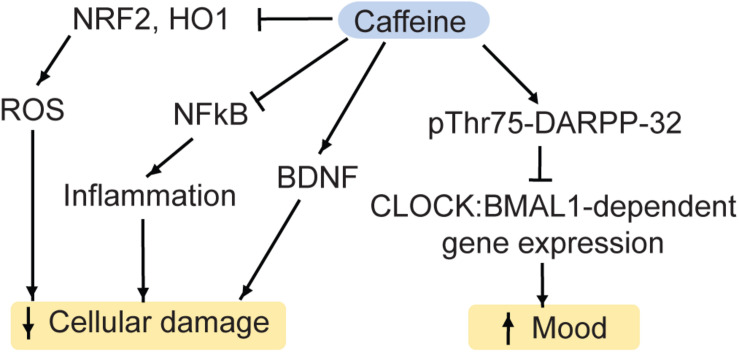
Caffeine regulates a variety of pathways, which impact on MDD. NRF2, nuclear factor-like 2; HO1, heme oxygenase 1; ROS, reactive oxygen species; DARPP-32, Dopamine and cAMP regulated phospho-protein 32kD; BDNF, brain-derived neurotrophic factor; CLOCK, Circadian Locomotor Output Cycle Kaput protein; BMAL1, Aryl hydrocarbon receptor nuclear translocation like protein 1.

The main molecular targets of caffeine are G-protein coupled adenosine receptors (A1, A2A, A2B, A3) (Ribeiro and Sebastio, 2010). Purinergic signaling has been generally associated with mood disorders (van Calker et al., 2019). At a first glance, adenosine receptors seem to regulate a complex web of pathways due to the fact that some receptor types have opposing functions. While the stimulation of A2AR increases symptoms associated with depression, A1R can promote rapid antidepressant effects (van Calker et al., 2019). This may be due to the fundamental classification of receptor types as A1R inhibiting cyclic adenosine monophosphate accumulation and subsequent activation of protein kinase A, and A2R stimulating it (van Calker et al., 1979, 1978). Among A2R, the A2AR has the highest affinity for adenosine (van Calker et al., 2019). In addition to regulating proteinase K activity, binding of adenosine to A1R inhibits the release of glutamate from the pre-synapse and reduces the postsynaptic excitability via potassium channels (van Calker et al., 2019). Furthermore, the activity of the A1R can be inhibited by the A2AR (Cunha et al., 1994), and the A2AR can interact with dopamine 2-receptors (Fuxe et al., 2007). This interplay makes it difficult to predict outcomes of purinergic stimulation or inhibition with pharmacological compounds such as caffeine on a receptor level. However, there is an integration of pathways downstream of protein kinase A, which integrates purinergic as well as glutamatergic and dopaminergic signaling: cAMP-regulated phosphoprotein Mr 32000 (DARPP-32) is a key signaling protein in the brain reward system, upon which a variety of signal transduction pathways converge (Yger and Girault, 2011; Figure 5). We recently showed that in mouse striatum, acute caffeine administration can ameliorate mood via the DARPP-32: Circadian Locomotor Output Cycle Kaput protein (CLOCK) pathway (Trautmann et al., 2020b). Specifically, caffeine increases the phosphorylation of Thr75 on DARPP-32 (Lindskog et al., 2002). Thr75 is necessary for DARPP-32 binding to CLOCK, a main regulator of circadian rhythms, which is implicated in mood disorders as well (McClung, 2007b, a). Importantly, DARPP-32 has been linked to mood disorders as well (Kunii et al., 2014). Mutation of Thr75 to alanine (T75A) inhibits DARPP-32 binding to CLOCK. Importantly, it also prevents the mood-elevating effects of caffeine, which occur in a diurnal manner (Trautmann et al., 2020b). Since caffeine affects CLOCK: Aryl hydrocarbon receptor nuclear translocation-like protein 1 (BMAL1) binding to chromatin, the caffeine-induced behavioral changes are likely mediated by gene expression changes, with the affected the gene products currently under investigation. Not only do CLOCK and BMAL1 influence the sleep-wake cycle and various circadian physiological functions (e.g., body temperature), but they also regulate the release of glucocorticoids (Landgraf et al., 2014; Mendoza, 2019). In turn, many CLOCK-regulated genes possess glucocorticoid responsive elements within their promoter regions (Kiessling et al., 2010; Landgraf et al., 2014). Hence, the caffeine-DARPP-32:CLOCK pathway may interact with stress-induced pathways as well, however this subject, too, is still under investigation.

Caffeine may also affect antidepressant-associated pathways independently of DARPP-32. For instance, in mouse hippocampus, acute caffeine can stimulate BDNF expression via the A1R-cAMP-CREB-BDNF pathway (Connolly and Kingsbury, 2010) or via the insulin receptor substrate 2 (IRS2)-phosphoinositide 3 kinase (PI3K)-Akt-pathway (Lao-Peregrín et al., 2017).

Moreover, acute treatment with caffeine reduces oxidative stress in rodents. Specifically, caffeine inhibits the markers of oxidative stress NRF2 and heme oxygenase 1 (HO-1), the pro-inflammatory cytokine NFκB as well as pro-apoptotic pathways (Hall et al., 2015; Endesfelder et al., 2017).

Human cross-sectional and prospective studies generally observed an inverse relationship between coffee intake and MDD-risk (Hall et al., 2015). Given various other bioactive compounds in coffee other than caffeine, these studies are by nature not conclusive about caffeine effects alone. To pinpoint the effects of caffeine, de-caffeinated coffee may be used as a control. In humans, the beneficial effects of caffeine occur at appropriate quantities (4–7 cups a day). However, a higher intake may actually impair mental health (Hall et al., 2015). In rodent models of depression, chronic caffeine improved motivational and cognitive deficits (Machado et al., 2020) as well as escape behaviors and measures of anxiety (Pechlivanova et al., 2012). Furthermore, chronic caffeine administration increased the resilience to chronic stress in rodents (Yin et al., 2015). While caffeine also appears to have fast-acting benefits on mood (Trautmann et al., 2020b) and synaptic plasticity of the brain reward system (Engmann et al., 2016), acute effects of caffeine need to be explored in further detail.

Besides caffeine, caffeinated beverages can contain other active substances, such as the nonselective phosphodiesterase and histone deacetylase-2 inhibitor theophylline (Barnes, 2013). In order to rule secondary effects due to these compounds, in human studies, caffeinated beverages should be directly compared with their decaffeinated counterparts or dissolved caffeine should be utilized.

In summary, caffeine appears to have several neuroprotective effects and may rapidly improve mood. However, studies using chronic and acute doses of caffeine are often difficult to compare. The psychostimulant effects of acute caffeine can impact on the interpretation of behavioral results gained after acute caffeine administration. Notably, the effects of acute caffeine are light-phase dependent in mice (caffeine improves mood only in the active phase), which needs to be taken into consideration for future investigations in rodents. The few human studies on the subject are difficult to interpret, perhaps because of the high baseline consumption of caffeine in the human population or due to other bioactive compounds in caffeinated beverages. Furthermore, there may an optimal dose of caffeine intake as well as common side effects such as high blood pressure. A variety of studies have described anxiogenic effects of caffeine under certain conditions (Charney et al., 1985; Bhattacharya et al., 1997). Nevertheless, the available research suggests that acute caffeine intake may rapidly improve mood and that caffeine may be a valuable research tool to potentially identify previously unknown antidepressant pathways.

## Conclusion – Nutrition an Depression: Researchers Still Have a Lot on Their Plate

Major depressive disorder is one of the most common mental illnesses worldwide. It is disabling not only to patients and their loved ones but represents a major economical burden to societies as well. Although there are various treatment options available, these often take weeks to fully function. In some patient cohorts, current antidepressants are not affective at all. For instance, in the STAR^∗^D trial, a major longitudinal study measuring antidepressant efficiency in more than 4,000 outpatients, the response rate to a 14-week treatement with selective 5-HT reuptake inhibitors was only 47% (Warden et al., 2007). It is therefore of great interest to find new approaches to treat or even help prevent MDD. In this review, we have discussed examples of dietary factors, which can assist in the therapy of MDD. These include essential nutrients such as B vitamins (B6, B9, B12), minerals (Zn^2+^, Mg^2+^) and PUFAs, which are frequently deficient in MDD-patients. Indeed, MDD may be a symptom of many dietary deficiencies. Hence, rectifying the diet can improve symptoms associated with monoamine signaling transmission, neural inflammation, HPA axis hyperactivity and oxidative stress. However, most research questions on this subject are still insufficiently addressed: Can chronic or acute supplementation of those dietary factors improve MDD-symptoms even in non-deficient patient populations? What are the underlying pathways beyond traditional hypotheses of MDD? In order to address these gaps in the field, mouse models of depression, such as the CVS-model (Labonte et al., 2017) should be chronically or acutely supplemented with various dietary factors and behavioral testing should be applied to observe behavioral improvements. Besides classical tests that address mood and anhedonia, circadian alterations need to be considered as well, since they are a substantial factor of MDD-symptomology. RNA-sequencing should be performed as an unbiased approach to identify altered gene products. Notably, research on animal models should be performed in their active phase, since acute behavioral effects can be masked by artificially measuring behavioral changes at a time, when mice are typically asleep (Trautmann et al., 2020b).

Caffeine has shown beneficial effects on mood in mice as well. As with the other dietary factors, effects of acute administration in human MDD-cohorts have been insufficiently documented. Given that dietary factors and caffeine are safe to administer, these studies should be feasible in the future. Special attention needs to be paid to separate patient cohorts that are regularly using vitamin supplements/caffeine and to take into consideration that this may introduce behavioral biases as well. Furthermore, studies should clearly state whether patients suffer from a nutritional deficiency before starting supplementation. Supplying nutrients beyond physiological requirements may have consequences distinct from a rescue of nutrient deficiency. Moreover, the timing of supplementation may be relevant. Data from animal studies suggest that certain interventions may reverse chronic stress effects with a supplementation at the end of the stressor. This may reflect best a scenario, when conventional rapid-acting antidepressant drugs are administered as well (Bagot et al., 2017). However, preventative measures may be a more sustainable approach to mental illness. Hence, the timing of supplementation, together with the assessment of the deficiency state of patients for multiple nutrients, should be a priority in future experiments. Furthermore, confounding factors present in many studies may lead to nutritional deficiencies and increased MDD risk as well. These include certain life style choices such recreational drug intake, a lack of exercise or unhealthy eating habits may both,. Future studies would have to control for those factors. Ideally, prospective studies randomly assign patients to placebo vs. supplement groups and measure MDD outcome in the future. This would allow for causal instead of purely correlative evidence. Experimenters should, however, assess deficiency levels in the beginning of the study. Unfortunately this has not been done in many previous prospective studies.

Another aspect that needs to be taken into consideration is a reduction of both, nutritional deficiencies and MDD symptoms by tertiary interventions such as antidepressants, exercise or psychotherapy. Furthermore, deficiencies may represent adaptive responses to risk factors for MDD such as inflammation and oxidative stress. However, independently of the cause of comorbid deficiencies, a rescue of MDD symptoms through supplementation remains intriguing. When studying the effects of supplements in patient cohorts, pharmacological interactions between supplements and medication (such as selective 5-HT reuptake inhibitors) may have to be taken into consideration.

When orally administered, supplements are likely to have a variety of systemic effects, all of which are insufficiently explored. For instance, they may affect the microbiome, liver metabolism or the HPA axis. Hence, brain physiology may be affected directly as well as indirectly. Approaches such as oral administration vs. i.p.-injection and microinfusion of supplements into various brain areas in MDD-mouse models will help to untangle the underlying mechanisms.

As several supplements converge onto the same pathways (inflammation, HPA-axis, DNA methylation), a variety of supplements may induce similar outcomes. This makes an interpretation of deficiencies more challenging, as a combination of deficiencies would have to be assessed. Equally, a combination of supplements may provide a similar, albeit unspecific, outcome, which may improve allover mental health. All these factors make studies on dietary factors in mental illness a complex undertaking.

This complexity is increased by the fact that dietary factors are unlikely to have specific effects for MDD. Instead, they may alter the risk for other mental illnesses such as BD and schizophrenia as well (Moustafa et al., 2014; Bozzatello et al., 2016; Ashton et al., 2019). We are not aware of supplements that benefit MDD while aggravating symptoms of mania or schizophrenia. Instead it is likely that certain dietary factors alter endophenotypes that are relevant to a spectrum of mental illnesses. For instance, DNA methylation is affected in MDD, but also in drug addiction (Brown and Feng, 2017) and Alzheimer’s disease (Qazi et al., 2018). Hence, altering the one-carbon metabolism through vitamins B9 and B12 may change the symptomology for all of these conditions. Accordingly, the relevance of dietary factors may have to be studied in the context of certain endophenotypes such as cognitive abilities, social interaction or psychosis, rather than the combination of symptoms that is known as MDD.

Ideally, personalized nutritional interventions would be adapted to other occurring stressors in patients’ lives. Based on currently available data, reversal or prevention of stress effects may benefit those patients most, which have been exposed to stress recently. In contrast, studies on nutrients’ effects on early life stress-induced symptoms are more sparse. Additionally, the interaction of nutrients with genetic components or environmental risk factors for MDD other than stress are largely unexplored as well.

Basic research on animals may identify more potent molecular targets regulated by dietary factors, which may ultimately be used to develop faster acting antidepressant drugs, which circumvent the side effects of currently available medications.

In this review, we have only discussed a few selected dietary factors. There are many other dietary compounds that have not been included in this review but may still have an effect on MDD. Vitamins B2 (riboflavin) and B3 (nicotinic acid) are highly relevant to metabolism as they are essential for the synthesis of flavin adenine dinucleotide (FAD) and nicotineamide adenine dinucleotide (NAD), respectively. NAD and FAD are not only key parts of the energy metabolism but they also participate in chromatin regulation (Berger and Sassone-Corsi, 2016).

Furthermore, the combination and timing of certain diets, such as ketogenic diet or intermittent fasting were not discussed here. Altogether, the field of nutrition research in psychiatry, while gaining more momentum in recent years, is still in its infancy.

## Author Contributions

JA wrote the initial draft of the manuscript. OE edited and rewrote the manuscript, checked, and corrected the references.

## Conflict of Interest

The authors declare that the research was conducted in the absence of any commercial or financial relationships that could be construed as a potential conflict of interest.
